# Commentary: The long-term psychological processing of an autism spectrum disorder diagnosis in parents

**DOI:** 10.3389/fpsyt.2026.1878275

**Published:** 2026-07-03

**Authors:** Hideki Muramatsu

**Affiliations:** Graduate School of Arts and Sciences, The Open University of Japan, Chiba, Japan

**Keywords:** autism spectrum disorder, children’s perspectives, family dynamics, family members’ suffering, parental mental health

## 1 Introduction

Martino et al. (1) is of significant academic value because it carefully examines parents’ long-term psychological processing of their child’s autism spectrum disorder (ASD) diagnosis through the lens of autobiographical memories. Identifying the prevalence of unresolved diagnoses and gender differences in the management of child-related caregiving tasks, efforts, and coping strategies is highly valuable. These findings highlight the need for parental support and frame ASD diagnosis not as a single event but rather a continuous process of meaning-making.

However, the discussion in this paper relies primarily on parental perspectives, while children’s subjective suffering and family dynamics receive relatively limited attention. Research clarifying parental suffering and support needs remains important and should not be overlooked. Simultaneously, as parent-focused studies accumulate, there is a risk that children’s suffering and the complex interactions within families will not receive sufficient examination.

A substantial body of research has examined the psychological burden and support needs of parents of children with ASD (2, 3). In contrast, research addressing parents’ own autistic traits and neurodevelopmental vulnerabilities remains limited and fragmented (4). Therefore, the potential influence of parents’ neurodevelopmental characteristics on family relationships and children’s suffering has not yet been sufficiently examined.

The purpose of this commentary is not to reject parent-centered ASD research but to highlight the need for a broader and more balanced framework that places greater emphasis on parental perspectives, children’s perspectives, and family dynamics.

## 2 Is it just the parents? — The need for a child’s perspective and family dynamics

In ASD research, parental perspectives often receive primary attention, while the tension and distress experienced by autistic children and their siblings (5) within the home, as well as asymmetries in family relationships, may not be sufficiently examined. This point is supported by recent studies emphasizing autistic children’s and youths’ own reports of quality of life and psychological well-being, including discrepancies between self-reported and parent- or caregiver-reported outcomes (6, 7). Future research could address these discrepancies by using methods tailored to the individual contexts and needs of children and adolescents with autism, rather than relying solely on a single method or informant. Such approaches may include self-report tools designed specifically for this population, as well as interactive and observational methodologies that capture their behavior and family interactions in relational contexts. Furthermore, children are not merely “diagnosed subjects” or passive recipients of support; they are also people whose psychological well-being may be shaped by family interactions and relational contexts (5). In addition, Martino et al. (1) themselves mention the limited or fragmented understanding of ASD, as well as the cognitive challenges observed in some parents, suggesting that educational background and the degree of understanding of the diagnosis can influence the integration of the diagnostic experience and subsequent adaptation. These discussions highlight the need to consider parental vulnerability not merely as a lack of individual effort but within a broader context that includes difficulties in understanding, cognitive vulnerability, and neurodevelopmental characteristics.

Some parents may not seek help, clinical consultation, or diagnosis because of stigma or broader cultural factors (8), and some may also have subthreshold autistic traits that do not meet formal diagnostic criteria (9). Recent evidence suggests that parental autism-related traits are associated with poor parental mental health. El-Bouhali-Abdellaoui et al. (10) reported that mothers of children in autism condition groups, including those with autism and autism traits, had poorer general mental health, and both fathers and mothers of children in autism condition groups scored significantly higher in anxiety/depression than parents of peers in the comparison group. In addition, Carmassi et al. (11) reported posttraumatic stress symptoms among parents of preschoolers who were first diagnosed with autism; they emphasized that acknowledging the possible presence of subthreshold autistic traits in some parents may be crucial for adapting interventions to the unique experiences of parents on the autism spectrum. Taken together, these findings suggest that hidden parental neurodevelopmental vulnerabilities should not be interpreted as parental inadequacy or blame but as factors that may increase susceptibility to trauma-related stress and chronic distress in the context of caregiving demands, stigma, and limited support.

## 3 Addressing the suffering of the whole family in ASD-related care

This argument is consistent with family systems approaches in ASD research (12), which emphasize the need for a balanced focus on both positive and negative factors involved in family functioning and for the inclusion of all family members in research methodologies. Rather than understanding the relationship between “caregivers” and “cared-for” as a fixed binary opposition, it needs to be understood as a relational process that can be transformed by interactions between family members and external peers, regardless of disability (13). This perspective can also connect with relational and recovery-oriented research frameworks that consider the possibility of mutual recovery, including among professionals (14). In this sense, this point is important as it reinforces the perspective that does not exclude any family member’s suffering from the analytical framework. The feelings of isolation and lack of support among parents discussed by Martino et al. (1) should be reconsidered not merely as individual parental problems but as issues that may also shape how children’s support is perceived and how support is unevenly distributed within family dynamics.

Building on existing family systems approaches in ASD research (12), it remains important to move beyond a primarily parent-centered accumulation of knowledge and to incorporate children’s perspectives and the perspective of the family as a whole. In research focusing on parents, responses may be shaped by child traits, which may introduce bias into the interpretation of parental experiences and vulnerabilities (9). Therefore, parental vulnerability should be approached cautiously and ethically, and more comprehensive research is needed that includes participants even without a formal diagnosis.

## 4 Conclusion

While preserving the important body of parent-focused research (4, 9–11) developed thus far, family research on ASD needs to move toward a more balanced incorporation of children’s perspectives, siblings’ experiences (5), and family dynamics. In particular, the issues of parental isolation and lack of support raised by Martino et al. (1) may provide an opportunity to reconsider not only parental adaptation but also the broader organization of support for families and the nature of support systems available to children. Furthermore, as the authors noted, understanding and integrating an ASD diagnosis is not merely a one-off event but a process that requires time, support, and a psychologically supportive clinical environment. In this sense, further development of this discussion requires a more comprehensive research framework that considers parental vulnerability, including cases in which no formal diagnosis has been made, while also incorporating children’s perspectives and the family as a unit. Recent longitudinal research on families of autistic children has also shown that parent mental health, child mental health, and family subsystems can be intertwined over time, supporting the need for longitudinal and multi-informant family studies (15). These findings indicate that clinical interventions may need to address not only individual parents or children, but also the parent-couple subsystem, where relevant, given its potential influence on both parental and child mental health. Further development of this discussion requires a comprehensive research framework that considers parental vulnerability, children’s and siblings’ perspectives, and the family as a unit, while considering multiple interacting factors such as autistic symptom severity, parents’ mental health histories, previous traumatic life events, and level of assistance required by the child (11). These arguments may help broaden and deepen the focus of ASD research on families. The present commentary may contribute to ASD family research by re-framing parental adaptation within a broader framework that also includes children’s perspectives and family dynamics.
